# Glossary of Computer‐Assisted Implant Surgery and Related Terms. First Edition

**DOI:** 10.1002/cre2.70148

**Published:** 2025-07-06

**Authors:** Adrià Jorba‐Garcia, Alessandro Pozzi, Zhuofan Chen, James Chow, Romain Doliveux, Yiu Yan Leung, Katsuhiro Maruo, Atiphan Pimkhaokham, Sofya Sadilina, Adam Siu, Kay Vietor, Feng Wang, Yiqun Wu, Man Yi, Bilal Al‐Nawas, Nikos Mattheos, ITI Network on CAIS, Peter Abrahamsson, Jonas Anderud, Sirida Arunjaroensuk, Marc Balmer, Michael Bornstein, Octavi Camps‐Font, Jinyan Chen, Aaron Chu Kai Yu, André Correia, Emilio Couso‐Queiruga, Roman Dittmar, Vincent Fehmer, Rui Figueiredo, Arndt Guentsch, Adam Hamilton, Holger Herweg, Martin Janda, Tim Joda, Ronald Jung, Kajorn Kungsadalpipob, Pascal Kunz, France Lambert, Alexander Lanis, Christel Larsson, Remo Malarik, Francesco Guido Mangano, Elias Messo, George Pelekos, Ali Murat Kokat, Clemens Raabe, Pimduen Rungsiyakull, Irena Sailer, Shakeel Shahdad, Andreas Stavropoulos, Franz Strauss, Keskanya Subbalekha, Jaijam Suwanwela, Baoxin Tao, Florian M. Thieringer, Daniel S. Thoma, Lin Jing Uei, Eduard Valmaseda‐Castellon, Daniel Wismeijer, Boon Kang Alvin Yeo, Xin Hui Yeo

**Affiliations:** ^1^ Department of Oral Surgery and Implantology, Faculty of Medicine and Health Sciences University of Barcelona Barcelona Spain; ^2^ Department of Clinical Science and Translational Medicine University of Rome Tor Vergata Rome Italy; ^3^ Department of Periodontics and Oral Medicine University of Michigan USA; ^4^ Department of Restorative Sciences Augusta University Augusta Georgia USA; ^5^ Department of Restorative Dentistry and Biomaterials Sciences Harvard School of Dental Medicine Boston Massachusetts USA; ^6^ Sun Yat‐sen University, Guanghua School of Stomatology Hospital of Stomatology; ^7^ Guangdong Provincial Key Laboratory of Stomatology Guangzhou China; ^8^ Dental Implant Surgery Centre Hong Kong‐SAR China; ^9^ Private Practice Mulhouse France; ^10^ Private practice, Neuenburg am Rhein Germany; ^11^ Oral and Maxillofacial Surgery, Faculty of Dentistry The University of Hong Kong; ^12^ Division of Prosthodontics and Oral Rehabilitation, Department of Oral Function and Restoration, Graduate School of Dentistry Kanagawa Dental University Yokosuka Japan; ^13^ Oral and Maxillofacial Surgery and Digital Implant Surgery Research Unit, Faculty of Dentistry Chulalongkorn University Bangkok Thailand; ^14^ Department of Oral and Maxillofacial Surgery, Faculty of Dentistry Chulalongkorn University Bangkok Thailand; ^15^ Clinic of Reconstructive Dentistry, Center for Dental Medicine University of Zürich Zürich Switzerland; ^16^ Private Practice Langen Germany; ^17^ Department of Implantology, Second Dental Center, Shanghai Ninth People's Hospital Shanghai Jiao Tong University School of Medicine Shanghai China; ^18^ Shanghai Key Laboratory of Stomatology Shanghai China; ^19^ State key Laboratory of Oral Diseases & National Center for Stomatology & National Clinical Research Center for Oral Diseases, West China Hospital of Stomatology Sichuan University Chengdu Sichuan China; ^20^ Department of Oral and Maxillofacial Surgery University Medical Center Mainz Mainz Germany; ^21^ Department of Dental Medicine Karolinska Institute Stockholm Sweden; ^22^ Faculty of Dentistry The University of Hong Kong Hong Kong SAR China; ^23^ Maxillofacial Unit, Halland Hospital Halmstad Sweden; ^24^ Oral Surgery Department Regional Hospital Halmstad Halmstad Sweden; ^25^ Department of Oral Health & Medicine, University Center for Dental Medicine Basel UZB University of Basel Basel Switzerland; ^26^ Department of Periodontology and Peri‐Implant Diseases Faculty of Medicine and Health Sciences of the University of Barcelona Barcelona Spain; ^27^ Department of Implantology, Second Dental Center Shanghai Ninth People's Hospital, Shanghai Jiao Tong University, School of Medicine Shanghai China; ^28^ Xpert Dental Group Hong Kong; ^29^ Centre for Interdisciplinary Research in Health (CIIS), Faculty of Dental Medicine (FMD) Universidade Católica Portuguesa Viseu Portugal; ^30^ Department of Oral Surgery and Stomatology, School of Dental Medicine University of Bern Switzerland; ^31^ Head Innovation Lab, Implantology Business Unit, Straumann AG Basel Switzerland; ^32^ Division of Fixed Prosthodontics and Biomaterials, and Dental Technical Laboratory University of Geneva Switzerland; ^33^ Department of oral surgery and implantology Faculty of Medicine and Health Sciences of the University of Barcelona Barcelona Spain; ^34^ Department of Periodontology Marquette University School of Dentistry Milwaukee USA; ^35^ Department of Restorative Dentistry and Biomaterials Science, Harvard School of Dental Medicine Harvard University Boston Massachusetts USA; ^36^ Division of Oral Restorative and Rehabilitative Sciences University of Western Australia Perth Australia; ^37^ VP, Implantology Business Unit, Straumann AG Basel Switzerland; ^38^ Department of Prosthodontics, Faculty of Odontology Malmoe University Malmö Sweden; ^39^ Clinic of Reconstructive Dentistry, Center of Dental Medicine University of Zurich Zurich Switzerland; ^40^ Department of Periodontology, Faculty of Dentistry Chulalongkorn University Bangkok Thailand; ^41^ Medprodent AG Switzerland; ^42^ Department of Periodontology and Oral Surgery, CHU of Liège University of Liege Belgium; ^43^ Division of Oral and Maxillofacial Implantology University of Chile School of Dentistry Santiago Chile; ^44^ Department of Prosthodontics, Faculty of Odontology University of Malmoe Sweden; ^45^ Innovation Manager, Innovation Lab, Implantology Business Unit, Straumann AG Basel Switzerland; ^46^ Department of Pediatric, Preventive Dentistry and Orthodontics I. M. Sechenov First State Medical University Moscow Russian Federation; ^47^ Private Practice Uppsala Sweden; ^48^ Deaprtment of Periodontology, Faculty of Dentistry The University of Hong Kong Hong Kong; ^49^ Department of Prosthodontics Istanbul Aydin University Istanbul Turkiye; ^50^ Prosthodontics, Faculty of Dentistry Chang Mai University Thailand; ^51^ Division of Fixed Prosthodontics and Biomaterials University of Geneva Switzerland; ^52^ Barts and the London School of Medicine and Dentistry Institute of Dentistry, Queen Mary University of London London UK; ^53^ Department of Periodontology, Faculty of Odontology University of Malmoe Sweden; ^54^ Universidad Autonoma de Chile Chile; ^55^ Department of Prosthodontics, Faculty of Dentistry Chulalongkorn University Bangkok Thailand; ^56^ Medical Additive Manufacturing Research Group Department of Biomedical Engineering University of Basel Basel Switzerland; ^57^ Department of Stomatology Ditmanson Medical Foundation Chia‐Yi Christian Hospital Chia‐Yin Taiwan; ^58^ Department of Oral Surgery and Implantology Faculty of Medicine and Health Sciences of the University of Barcelona Barcelona Spain; ^59^ Private Practice, Ellecom The Netherlands; ^60^ Periodontology, Faculty of Dentistry National University of Singapore Singapore

**Keywords:** computer‐assisted implant surgery, digital dentistry, guided surgery, implantology

## Abstract

The rapid development of computer‐assisted implant surgery (CAIS) and the respective research and clinical applications have necessitated a standardization of the terminology related not only to different devices, but also the different steps involved, surgical and presurgical procedures. The present glossary was introduced at the 1st International Team for Implantology Symposium on Computer‐assisted Implant Surgery, based on the collective work of clinicians and researchers with deep understanding and experience in these technologies. The glossary was further refined and revised through the structured input of a large group of global experts within clinical application, research, and education of CAIS. The glossary includes 98 terms organized in 5 domains, aiming to clarify ambiguity and propose some standard nomenclature in the service of clinical practice, research but also development of new devices, protocols, and approaches.

## 1 Preface

Computer‐assisted implant surgery (CAIS) is encompassing an array of different technologies and protocols and is gaining importance in the daily clinical practice, while the volume of related research is also increasing fast. The rapid development of these novel technologies and the multiple research and clinical approaches have necessitated a standardization of the terminology related not only to different devices, but also the different steps involved, surgical and presurgical procedures. Continuously optimizing and updating the terminology could help both clinicians and researchers to communicate, better describe and define devices and interventions, while correctly interpreting research results and clinical outcomes. To that end, the 1st International Team for Implantology (ITI) Symposium on Computer‐assisted implant surgery, introduced this glossary of terms, as based on the collective work of clinicians and researchers with deep understanding and experience in these technologies.

This glossary includes a set of terms and their definitions to clarify ambiguity and propose some standard nomenclature in the service of clinical practice, research but also development of new devices, protocols and approaches.

Several examples of such glossaries in different disciplines, have been extremely valuable in the past, such as the Glossary of Prosthodontic Terms (The Glossary of Prosthodontic Terms 2023: Tenth Edition) devised and updated by the Academy of Prosthodontics or the Glossary of Digital Dental Terms (Glossary of Digital Dental Terms, 2nd Edition: American College of Prosthodontists and ACP Education Foundation 2021) from the American College of Prosthodontics. It is certainly not the aim of this glossary to replace such valuable works as the above‐mentioned, which have served as inspiration to this study. However, there was a void of critical terminology in the surgical domain of the digital workflow, while some of the currently used terms were often met with generic definitions with little relevance to the computer‐assisted implant surgery application. Previous papers initiated by the ITI have introduced some fundamental terminology (Jung et al. 2009), reflecting the current developments however would require a renewed and wider effort.

This initiative was undertaken with twofold aims: first to support clinicians and researchers who enter the field of CAIS with a clear and concise organization of the essential terminology in the clinical workflow, as well as define terms currently missing from other wider glossaries. Second, with the help of a wide panel of experts, to clarify and streamline critical terms which are currently ill‐defined or frequently misunderstood and misleading.

## 2 Methods

A core team of experts with both clinical workflow as well as current literature in CAIS (A.G.J., N.M., S.S., A.P., and B.a.N.) conducted a literature review in PubMed aiming to identify terms critical for describing procedures and outcomes with guided implant surgery. Terms were included by consensus only. The core team compiled a preliminary draft, organizing 58 terms in 4 groups, which was then circulated and commented upon by a wider group of selected experts (main authors) between September and November 2024. The final draft was produced by consensus of all main authors after a dedicated day of discussion at the side of the 1st ITI Symposium on CAIS, 20–21 November 2024, Chulalongkorn University, Bangkok, Thailand, and included 96 terms organized in 5 groups. This version was further revised by 44 clinicians and researchers, identified as global experts in the field (ITI Network on CAIS, co‐authors, credited in Acknowledgments section). This final consultation period occurred between November 2024 and March 2025 and resulted in several refinements and the addition of 2 more terms. The final version of the Glossary with 98 terms/5 groups was endorsed in its present form by all main authors and experts (co‐authors) named in the Acknowledgments section.

## 3 Discussion

**Figure 1:**
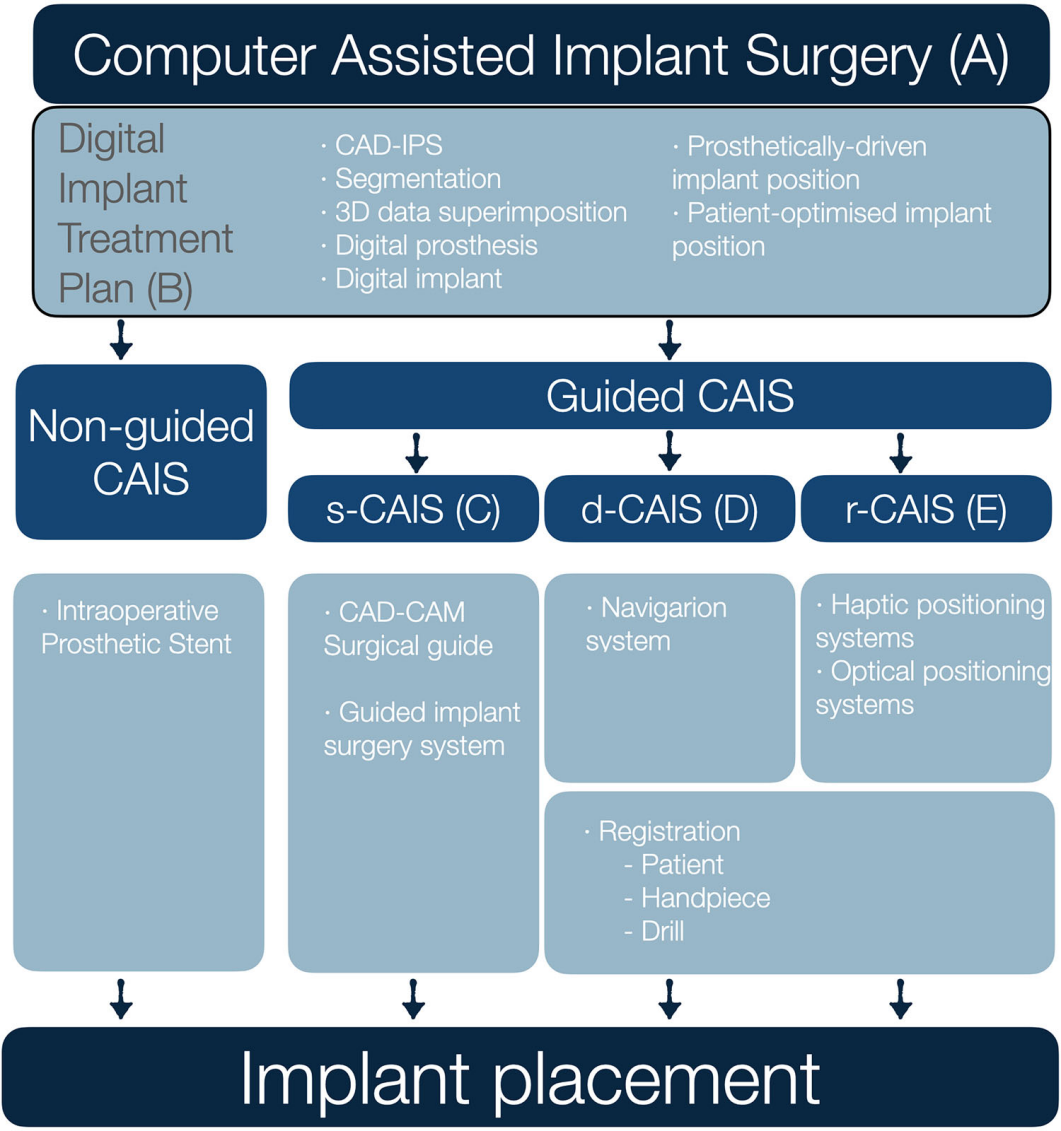
Overview of the organization of computer‐assisted implant dentistry, with selected terms arranged against common workflows. The corresponding section of the Glossary for each of these domains is provided after the section in parenthesis (A–E).

From the start, it was well understood that a glossary in such a rapidly developing field could only be a “work in progress” and the authors engaged with the terms accepting that perfection was elusive in such a fast‐growing domain of science. New devices are introduced accompanied by new terms, often contradicting previous ones or terms in other similar domains of medicine and science. In this glossary, the aim of the authors was to provide clear organization of the terminology matched against the critical steps of the workflow, while at the same time maintaining the definitions as close as possible to the established ones where available (Figure 1). For example, in the case of the synonyms “computer‐aided” and “computer‐assisted” the authors chose to maintain the widely established norms and keep the first as is in “computer‐aided design” and the latter as is in “computer‐assisted implant surgery.” In other cases where controversial or ill‐defined terms were encountered, a debate was necessitated to reach a consensus or majority, while at the same time acknowledging the advantages and disadvantages of every choice. An effort was conducted to interpret generic terms in relevance to the practice of implant dentistry where necessary.

## 4 Guided Versus Non‐Guided CAIS

Although “guided implant surgery” or CAIS are often perceived as describing the surgical placement of dental implants, it was clear to the authors that the term describes workflows much wider, which include essential procedures and devices at multiple steps before the actual surgical intervention. The term “computer‐assisted” was then selected as opposed to “computer‐guided,” as the first would be open to imply the use of digital technology in procedures preceding the surgery, while the latter might be more suggestive of active “guidance,” which mainly focuses on the surgical intervention. The ability to surgically place a dental implant with accuracy has no meaning outside of a comprehensive digital treatment plan, which in turn is not possible without the collection of the adequate 3‐dimensional diagnostic data, the evidence‐based understanding of sound restorative and surgical design principles and the proper process in a computer‐aided design – implant planning software (CAD‐IPS). Recognizing this, the authors defined CAIS as a workflow, incorporating essential diagnostic and treatment plan steps, to digitally identify the optimal implant position. The authors firmly believe that this planning process results in the digital implant treatment plan should be the current minimum standard of care for implant therapy. Following this, different approaches can be utilized for the surgical placement of the implant: from conventional freehand (non‐guided CAIS), to static, dynamic, or robotic placement (guided CAIS) (Figure 1). The authors maintained the non‐guided CAIS, acknowledging that implants with a proper digital treatment plan can be placed freehand by properly trained clinicians where indicated. A clear distinction was made, however, when the implant placement is directed by technologies with active guidance or real‐time navigation support, which was then described as “guided CAIS.” The term “reference‐guided” was considered for the non‐guided CAIS workflow, to reflect that analog‐produced intraoperative prosthetic stents could serve as reference “guiding” the clinician in the implant placement. However, it was widely considered that such a use of the term would essentially lower the threshold of what “guidance” truly implies. In short, the authors consider non‐guided CAIS (digitally planned – freehand placed) as the minimum essential standard in implant therapy today and acknowledge that it is suitable for conventional workflows and cases of lower complexity. Nevertheless, clinical protocols with complex workflows such as implant placement in extraction sockets, immediate loading, flapless placement, multiple implants, or fully edentulous patients would greatly benefit from or only be possible with the use of guided CAIS workflows.

### 4.1 “Digital” Versus “Virtual”

Very early in the process, it became clear that these two terms – both frequently encountered in the CAIS workflow and related terminology – were typically treated as synonyms. The truth, however, is that they define distinctly different concepts: a “digital” object is one created by means of digital technology and existing within a digital space or environment, whereas a “virtual” object, simulated or projected in a physical or simulated space. The distinction led to an important question in the context of the digital treatment plan: Can a “virtual” object (e.g., virtual implant) exist that is not at the same time also “digital”? Well, the answer was that it can, or at least it has so been in the past. Many co‐authors recalled a time when implant treatment plan was performed using transparent sheets depicting implant shapes at various magnifications. These transparencies were then manually superimposed onto the panoramic or periapical radiograph allowing clinicians to select the right implant and draw it with a pencil in the appropriate position in the 2D anatomic representation of the patient. This was a virtual implant a 2D format, but was not digital. Nevertheless, in modern implant dentistry, a virtual tooth, implant or prosthesis will inherently be a digital one. Thus, the authors have accordingly refined the terminology to clarify when “digital” and “virtual” can be used interchangeably and when not.

### 4.2 “Prosthetically Driven” Versus “Patient‐Optimized” 3‐Dimensional Implant Position

The authors strongly encourage the current paradigm of prosthetically driven implant placement, where implant positioning is part of designing a system and follows a top‐down approach: starting with the design of the optimal prosthesis (Pedrinaci et al. 2024), followed by design of the corresponding supracrestal complex (Mattheos et al. 2021) and finally determining the implant size and position which seamlessly serves the above designs based on best available evidence. Thus, it was deemed essential to define the term “prosthetically driven 3D implant position,” as its identification is of paramount importance in the digital treatment plan.

While compatibility with the ideal prosthesis is essential, other important factors must be considered when defining the implant position. These include, among other proximity to vital structures, likelihood to achieve primary stability and osseointegration, securing proper flap closure and tissue healing, as well as adequate soft tissue thickness and volume. Although essential, the prosthetically driven position alone might not always automatically satisfy all these critical parameters. In such cases, the digital treatment plan allows for testing alternative prosthetic designs, implant types, dimensions, and positions to identify the position where all essential conditions would be best met. Thus, another term was defined as “patient‐optimized” implant position, to define the position which entails all essential requirements including – but not limited to – the prosthesis design. The patient‐optimized position should be the end‐point deliverable of the digital treatment plan, and it describes the virtual implant position which has the highest likelihood of fulfiling all the necessary requirements, including the proper support of the prosthesis design. There was a long debate as to which terms best expresses this position. Finally, “patient‐optimized” was selected, as it emphasizes two important aspects:

1. individualization: this position is tailored to each patient, considering many patient‐specific parameters ranging from anatomic structures to patient's needs and individual risk assessment.
2. Optimization: the term “optimized” is suggestive of the essential decision‐making process from the clinician, who needs to carefully consider all patient, anatomic, functional, and esthetic parameters directed by the best available evidence and patient needs.

Identification of the patient‐optimized position remains the responsibility of the clinician. As the technology of implant surface, fixture, and restorative designs advances, the potential to achieve a patient‐optimized implant position which is at the same time prosthetically driven is currently very high.

### 4.3 “Top‐Down” Versus “Bottom‐Up” Digital Implant Treatment Plan

All authors agreed that prosthetically driven design requires a “top‐down” approach (Puisys et al. 2023), which implies the use of the virtual prosthesis as the starting point for the digital treatment plan. It was, however, apparent that most of the established CAD‐IPS followed the opposite direction of the workflow, starting the design “bottom‐up” and building on top of the virtual implant. In most CAD‐IPS restorative components cannot be added unless the virtual implant is in place, and there is no automated connection between the virtual prosthesis and the virtual implant/abutment or other components. This limitation highlights the need for CAD‐IPS workflows that automate the process from a top‐down perspective, allowing the virtual prosthesis to guide implant positioning. Achieving this would require a fundamental paradigm shift from the current established norms followed by many commercially available CAD‐IPS systems.

## 5 Accuracy, Trueness, and Precision

The latest directive of the International Standardization Organization (ISO 5725) defines “accuracy” of measurements as composed of two essential components: trueness and precision (International Standardization Organization). These components account for different types of errors; trueness reflects random errors while precision relates to systemic errors. Applying this principle to CAIS can be confusing, as most of the clinical studies assessing accuracy of implant placement have in fact only reported trueness. This might not be entirely surprising, given that ISO 5725 directive states that “the term accuracy was at one time used to cover only the one component now named trueness,” but it was later broken down to two elements to account for both random and systemic errors. Further complicating its application in CAIS is the fact that clinical trials can only assess trueness, while assessment of precision would require reproducible implant placement, which is possible only in simulation studies. Despite the discrepancy with the current status quo in clinical studies, it was deemed essential to streamline the terminology with the widely applied standards of ISO, thus the terms Accuracy, Trueness, and Precision were adjusted accordingly. In the future, clinical and simulation studies might need to clearly define which one of the three aspects is being assessed and how they are measured.

## 6 Registration Versus Calibration

One of the most confusing concepts in the domain of dynamic and robotic CAIS was the distinction between “calibration” and “registration.” The authors realized early that these two very important terms were often used interchangeably or inconsistently to describe procedures during the set‐up for navigation or robotic surgery. In fact, these two terms refer to fundamentally different procedures. Registration implies the establishment of a 3‐dimensional space by the computer of a navigation system and defined by a system of X–Y–Z coordinates. To track every object which moves within this space, its shape and position have to “register” with the system and remain thereafter attached to a tracker. Thus, before every navigation or robotic surgery, a registration of the patient, the handpiece and each individual drill takes place, which thereafter allows the system to track these objects in real time.

Calibration on the other hand, describes an entirely different process, which aims to ensure that the values returned by a system are in agreement with what is defined as ground truth. For example, in a CBCT machine, calibration ensures that the digital representation of a tooth dimensions accurately reflects its real‐world dimensions and position, allowing precise measurements. Every digital device and software requires calibration, which however is subject to industrial specifications and not a process that usually involves the clinician. Calibration usually includes a well‐defined, universal, or individual (serial‐numbered) geometric object that is scanned by the device to be calibrated, and the obtained output is compared to a known master file. To consider the calibration successful, outputs need to exactly match the dimensions of the respective geometric object or to deviate no more than the tolerance allowed by the specifications. Defective registration or calibration will both result in systemic errors and reduced accuracy, but through very different mechanisms. Thus, the authors have taken a clear position when defining these two terms.

## 7 Data Registration Versus Superimposition

During the discussion about registration, the term “data registration” was proposed to describe the superimposition of the DICOM and STL files in the CAD‐IPS during the digital treatment plan. This would be true since a “master” 3‐dimensional space defined by coordinates is created in the CAD‐IPS, in which the files have to be aligned to. In the current digital treatment plan workflow, the coordinates of the CBCT which derive from the actual positioning of the patient will be used as “master,” into which the surface scan file will be then “registered.” Since 3‐dimensional files always come with their own system of coordinates, when two such files are aligned there will always be one of the coordinates system used as “master,” even if not directly selected by the user. Even when digital files are overlayed manually based on surface morphology similarities and/or digital landmarks, the final outcome will be defined by one system of coordinates. Thus, the term 3d data registration was preferred to commonly used synonyms such as it best describes the process. Among the various terms discussed, mesh alignment data orientation, superimposition or overlay were noted as commonly used synonyms. The terms “fusion” and “merging” were considered misleading, as they would imply that the two data sets are permanently combined/joined into one file or object.

This first edition of the glossary does not aim to be exhaustive, but seeks to define critical terms, address common misconceptions and clarify terminology essential for CAIS workflows. Consequently, some technologies or procedures may not yet be included and certain entries may evolve through revision and refinement.

At the same time, effort was taken to define the terms in the context of CAIS workflow, thus at time more general and overriding terms were adopted and adjusted from other glossaries. The authors would like to initiate a collaborative discussion, among academics, clinicians, and researchers to further expand and enhance this glossary. Future updates will be carried out under the auspices of the ITI to ensure continued relevance and accuracy. All constructive suggestions are welcome and will be carefully reviewed for inclusion in future editions. Thus, the first edition of the Glossary of Computer‐Assisted Implant Surgery and related terms has been collectively created by a large group of experts and is now available for clinicians and researchers to refer and contribute to.

## 8 Explanatory Notes

### 8.1 Entries

- All terms are presented in bold, followed by their definition in regular type.
- All terms are organized in 5 sections, matched against essential steps of the workflow. When terms are grouped, the overriding term precedes, followed by terms reflecting subcategories. Terms within each section are arranged alphabetic order.
- Commonly used or recommended abbreviations, *“Abr,”* are proposed to follow the main term. Synonyms of a term are listed after the definition, introduced by the label “*Syn.*” Terms commonly confused are marked with “Not to be confused with ‐”
- When a definition is based on or modified from a definition published elsewhere, the respective source is cited in the end of the definition. Definitions adjusted from other sources have been usually simplified/modified for relevance with specific use within CAIS.
- Each term in each section is numbered for quick reference.

## 9 Glossary

**A. Overview and Organization**

**1. Computer‐Assisted Implant Surgery**

*Abr*: CAIS

The use of digital technologies, software and devices to plan the patient‐optimized 3D position of the dental implant, which will then be surgically placed by means of

a. non‐guided (ng‐CAIS),

b. static (s‐CAIS),

c. dynamic (d‐CAIS), and

d. robotic (r‐CAIS).

*Syn*: Computer‐Aided Implant Surgery

Not to be confused with: Computer‐assisted implant placement (CAIP), computer‐guided implant surgery

**2. non‐guided Computer‐Assisted Implant Surgery**

*Abr*: ng‐CAIS

The use of digital technologies, software and devices to plan the patient‐optimized 3D position of the dental implant, with the osteotomy and the placement to be conducted freehand, with no active guidance. The surgeon may use anatomic landmarks or measurements, as well as directional analog aids (vacuum form stents or “suck‐downs” based on wax‐up etc. (see *intraoperative prosthetic template B20*) to help identify the planned implant position in the patient's oral cavity.

*Syn*: mental‐, freehand‐, brain guided ‐ CAIS

**3. static Computer‐Assisted Implant Surgery**

*Abr*: s‐CAIS

The use of digital technologies, software and devices to plan the patient‐optimized 3D position of the dental implant and the consequent use of CAD/CAM surgical guides (see C4) to assist the implant osteotomy and accurate placement of the implant in the planned position and/or relevant modifications to the anatomy of the surgical site.

**4. dynamic Computer‐Assisted Implant Surgery or Navigation CAIS**

*Abr*: d‐CAIS

The use of digital technologies, software and devices to plan the patient‐optimized 3D position of the dental implant and the consequent implant osteotomy and placement of the dental implant in the planned position supported by a navigation system which tracks continuously and in real‐time handpiece and patient tracking arrays to determine their precise position and orientation in a common coordinate frame during the surgery. The position of the drills and handpiece in relation to the patient's anatomy is presented on a digital display together with relevant information about the drilling as angular and linear deviation from the planned position and the progression of the drill along the anatomic recipient site.

*Syn*: Navigation systems, real‐time navigation, Dynamic Navigation.

**5. robotic Computer‐Assisted Implant Surgery**

*Abr*: r‐CAIS

The use of digital technologies, software and devices to plan the patient‐optimized 3D position of the dental implant and the consequent implant osteotomy and placement of the dental implant in the planned position supported by robotic technology with varying levels of automation ranging from task assistance to autonomous robotic execution under human supervision/control.

*Syn*: Robotic implant surgery

**6. Hybrid‐guided CAIS**

*Abr* **:** h‐CAIS

The combined use of two or more guided CAIS approaches (s‐CAIS, d‐CAIS, or r‐CAIS) during the same implant surgery procedure, where different surgical steps are performed using different guidance methods. At present, only few clinical trials have reported on the use of combined s‐ and d‐CAIS.

**B. General Terms and Treatment Plan**

**1. 3D Data Registration**

The process of alignment and orientation of files with 3‐dimensional anatomic representations or 3 ‐dimensional medical imaging with a specific system of 3‐dimensional coordinates. Registration of one or more intraoral surface scans (e.g., STL [Standard Tessellation Language], PLY [Polygon File Format], etc.) and DICOM files from Cone Beam Computer Tomography (CBCT) to the 3‐dimensional coordinates maintained in the CAD‐IPS commonly takes place during the digital implant treatment plan.

*Syn*: 3D data superimposition, ‐overlay – matching, mesh alignment,

**2. 3D Data Segmentation**

The process of extracting or isolating specific anatomic structures such as the jaws, zygomatic bone, mandibular canal, maxillary sinus, teeth or roots from a set of DICOM (Digital Imaging and Communication in Medicine) files during the digital treatment plan. Segmentation usually involves conversion of DICOM data into a surface or object, usually an STL (Standard Tessellation Language) file. It could be done manually on CAD software or using automations and algorithms based on gray‐value thresholds driven by artificial intelligence.

**3. Augmented Reality**

*Abr*: AR

Technologies which superimpose digital content (images, sounds, data, etc.) onto the real world, typically through a screen, such as a smartphone, tablet, head‐up display (HUD) or AR glasses. AR adds layers of information on top of the real‐world view but doesn't necessarily integrate or interact with it.

Adjusted from the US Food and Drug Administration (US Food and Drug Administration n.d.).

**4. Accuracy (of implant placement)**

The closeness of the actual implant position to the planned one, which involves a combination of random components and a common systematic error or bias component. Thus, accuracy is composed of two parameters: trueness and precision.

Adjusted from ISO 5725‐1:2023 (International Standardization Organization n.d.).

**5. Precision (of implant placement)**

The closeness of more than one consecutive measurement of the actual implant position to each other. Typically, precision reflects the clustering of consecutive measurements of trueness under identical implant placement conditions, thus It can be assessed through simulation studies only. Precision expresses the consistency or repeatability of measurements, showing how closely grouped the results are to each other, regardless of whether they are close to the true value.

Adjusted from ISO 5725‐1:2023 (International Standardization Organization n.d.).

**6. Trueness (of implant placement)**

The closeness of the actual implant position to the planned one. Typically assessed by measuring the arithmetic mean of the deviation of the actual from the planned position in a large number of placements. Deviation is commonly assessed at (a) the implant platform and b) apex (in mm) and the deviation of (c) the implant axis (angle, in degrees). It can be assessed through both simulation (in vitro) and clinical studies. Adjusted from ISO 5725‐1:2023 (International Standardization Organization n.d.).

**7. Calibration**

The process of assessing or adjusting the output of a device in agreement with the value of the applied standard, within a specified margin of accuracy. For example, calibration of a CBCT ensures that the displayed dimensions of the anatomic structures correspond to the actual ones, allowing precise measurements within the specified accuracy of the system. Several devices within CAIS workflows require calibration, from the CBCT, IOS, CAD‐IPS, CAM machines and the navigation systems of d‐CAIS and r‐CAIS. Calibration is a process commonly performed by manufacturers or certified experts and not the end users.

Not to be confused with: Registration (see D12).

Adjusted from National Institute of Standards and Technology, Gaithersburg, USA (National Institute of Standards and Technology [NIST]).

**8. Computer‐Aided Design and Computer‐Aided Manufacturing**

*Abr*: CAD‐CAM.

Digital technologies which involve the virtual design (CAD) of an object and subsequently the manufacturing process (CAM) to physically create the designed object.

Adjusted from the Glossary of Prosthodontic Terms 10th edition (The Glossary of Prosthodontic Terms 2023: Tenth Edition).

**9. Computer‐Aided Design Implant Planning Software**

*Abr*: CAD‐IPS

CAD software specifically developed for the digital plan of dental implant treatment including the ability to perform detailed measurements. It can be (a) ***complete,*** when allowing for the design of the prosthesis, supracrestal complex and implant position in relation to anatomic landmarks, bone and oral mucosa or (b) ***partial,*** when it allows the planning/designing of only some of the above elements (e.g., the implant position, abutment design, etc.).

*Syn*: treatment planning software, Implant planning software

**10. Digital Implant**

The virtual dental implant when created by CAD technologies and depicted in relation to patients' anatomy by means of previously obtained 3D images (i.e., cone‐beam computerized tomography and surface scan).

**11. Digital implant treatment plan**

The design of the virtual prosthesis, prosthetic components and implant type and position in appropriate relation to the patient's anatomic structures (occlusion, soft and hard tissue), created by digital technologies and depicted in previously obtained 3D images (i.e., Cone‐beam computerized tomography and surface scan).

*Syn*: *Digital implant plan, virtual treatment plan (VTP), virtual implant plan*

**12. Digital Landmark**

A point depicting an anatomic landmark in a 3‐dimensional digital file, the corresponding point of which can be identified in another 3‐dimensional file or medical image. Digital landmarks (e.g., teeth cusps) are typically used as reference or fiducials in the process of 3D data registration (see B1) to align different 3D digital files in one system of X, Y, Z coordinates.

**13. Digital Prosthesis**

The virtual dental prosthesis when created by CAD technologies and depicted in relation to patients' anatomy by means of previously obtained 3D images (i.e., Cone‐beam computerized tomography and surface scan). Commonly used for the purpose of digital treatment planning, also referred to as “digital wax‐up” or “mock‐up,” “digital diagnostic tooth arrangement,” when placed in optimal position in relation to digital representation of the remaining dentition.

*Syn*: Virtual prosthesis, digital wax up, digital setup, “digital diagnostic tooth arrangement,” mock‐up of prosthetic virtual planning.

**14. Landmark**

An anatomical or artificial point in the patient's oral cavity, the corresponding point of which can be identified in 3‐dimensional digital file or a medical image. In d‐CAIS workflows, landmarks are used as fiducials to perform markerless pair‐point registration (see D20).

**15. Mixed Reality**

*Abr*: MR, XR

Technologies which seamlessly integrate digital objects into the real world. MR combines both real and virtual elements in ways that they can interact with each other, creating a more immersive experience than AR. MR enables a higher level of interaction and environmental awareness.

*Syn*: extended reality, merged reality

**16. Patient‐optimized 3D implant position**

The virtual 3‐dimensional implant position which in the highest likelihood fulfils all essential requirements, including the proper alignment with the prosthesis design, safety‐distance from critical anatomical structures, primary stability, essential conditions for healing of the soft and hard tissue and if needed, allows for simultaneous or staged tissue augmentation procedures.

*Syn*: ideal‐, personalized‐, optimal‐ implant position, Bio‐Restorative Implant Position (Pedrinaci et al. 2024)

**17. Photogrammetry**

A digital technology applied in CAIS by utilizing multiple optical cameras to capture a series of 2D images of specially designed trackers firmly attached on dental implants in the mouth. Utilizing triangulation and mathematical relations the system can then create a precise 3D model of the implants' positions and angulations in relation to the patient's anatomy. It can be conducted by:

a. extraoral devices

b. intraoral devices or

c. navigation (d‐CAIS) systems.

**18. Prosthetically driven 3D implant position**

The 3‐dimensional implant position which derives from the prosthetically driven digital treatment plan (see B19) and which is fully aligned with the design of the prosthesis and the supracrestal complex, as directed by patient anatomic conditions and best available evidence.

**19. Prosthetically driven digital implant treatment plan**

The digital implant treatment plan which utilizes the virtual prosthesis as a reference for the design of all other component types, dimensions, and positions. The virtual design of the prosthesis which fulfils esthetic, function, and oral hygiene requirements precedes, followed by the design of the corresponding supracrestal complex (Mattheos et al. 2021) in accordance with best available evidence and individual anatomic conditions. Finally, implant position, type and dimensions are identified for optimal support of the aforementioned virtual designs.

*Syn*: top‐down treatment plan, restorative‐driven treatment plan, Bio‐Restorative Implant planning (Pedrinaci et al. 2024)

**Prosthetic Template ‐**

**20. Intraoperative**

A template, allowing the visual superimposition of a prosthesis shape (shell) onto an edentulous site during implant surgery. Typically, analog manufactured after a conventional “wax‐up” or tooth “set‐up,” such templates have been frequently used in ng‐CAIS protocols to help the surgeon to intraoperatively visualize the margins/shape of the planned prosthesis and consequently define the final position of the implant.

*Syn*: surgical stent, conventional surgical guide, surgical splint, reference guide, analog guide

**21. Radiographic**

Analog‐manufactured template typically replicates a partial or full denture with precise fit on the adjacent teeth or mucosal surface and the planned position of the teeth as in the planned restoration. The template includes several radio‐opaque markers (at least 3).

*Syn*: radiographic stent, radio‐opaque prosthetic template/guide

**22. Virtual implant**

The projection of a dental implant in appropriate relation to the anatomic structures of the patient – Synonym to Digital Implant (see B10) when created by digital technologies and depicted in previously obtained 3D images (i.e., Cone‐beam computerized tomography and surface scan).

**23. Virtual prosthesis**

The projection of a dental prosthesis in appropriate relation to the patient's anatomic structures (occlusion, soft and hard tissue) ‐ Synonym to Digital Prosthesis (see B13) when created by digital technologies and depicted in previously obtained 3D images (i.e. Cone‐beam computerized tomography and surface scan).

*Syn*: mockup, setup

**24. Virtual Reality**

*Abr*: VR

Technologies which create a 3‐dimensional immersive, digital environment, isolating the user from the real world, with or without interaction. The digital environment can typically be accessed through a VR headset.

Adjusted from US Food and Drug Administration (US Food and Drug Administration n.d.).

**C. static Computer‐Assisted Implant Surgery**

**1. 3D printing**

Additive technologies of creating three‐dimensional objects previously designed by CAD by adding material layer by layer.

*Syn*: 3D rapid prototyping

Adjusted from the Glossary of Prosthodontic terms. Tenth edition (The Glossary of Prosthodontic Terms 2023: Tenth Edition).

**2. Anchor pin**

Metallic components are used intraoperatively to temporarily anchor and stabilize the surgical guide in the bone. Anchor pins are typically placed through specially designed channels in the surgical guide and are inserted in the bone after narrow drills have prepared the recipient site.

**3. Bone Offset**

**Figure 2:**
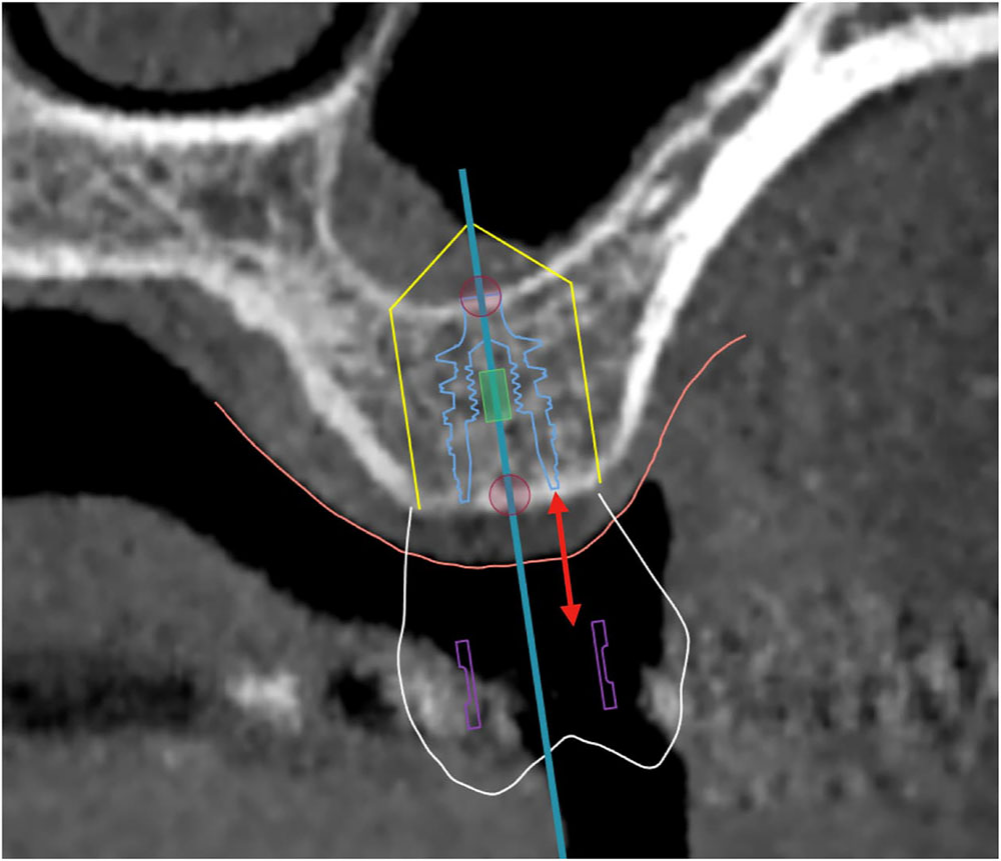
Bone offset (C3): The distance (red arrow) from the apical end of the sleeve (purple) to the level of the marginal bone or shoulder of the implant (blue).

The vertical distance from the apical end of the sleeve of the CAD/CAM surgical guide to the level of the marginal bone (Figure 2).

*Syn*: Guide sleeve offset

Not to be confused with: Gingival height

**4. CAD/CAM Surgical guide**

Computer‐aided designed and manufactured (CAD/CAM) device aiming to guide the osteotomy and the placement of a dental implant in the planned position or be used to guide other modifications of the anatomy, such as ostectomy, gingivectomy, sinus surgery, etc. This device is used temporarily in the patient's oral cavity during surgery with or without fixation elements.

Not to be confused with: intraoperative stent – splint

**5. Tooth‐supported surgical guide**

A CAD/CAM surgical guide supported solely by natural teeth, and/or fixed dental‐ or implant‐borne tooth‐reconstructions.

**6. Bone‐supported surgical guide**

A CAD/CAM surgical guide supported solely by bone (after a full flap reflection). Can be with or without fixation elements.

**7. Mucosa‐supported surgical guide**

A CAD/CAM surgical guide supported solely by mucosa (e.g., in fully edentulous patients) with or without fixation elements.

**8. Hybrid‐supported surgical guide**

A CAD/CAM surgical guide supported by more than one tissue. Examples: Surgical guide in Kennedy class I edentulism supported by teeth and mucosa; or mucosa supported guides in fully edentulism combining also fixation elements, such as bone anchor pins.

**9. Stackable surgical guide**

A multi‐layered CAD/CAM surgical guide, used commonly in complete‐arch implant surgery to sequentially guide bone reduction, implant placement, and prosthetic positioning with high precision. A stackable guide allows different parts to be stacked and removed in phases, ensuring accurate execution of each surgical step while maintaining prosthetically driven implant placement.

**10. No‐sleeve surgical guide**

**Figure 3:**
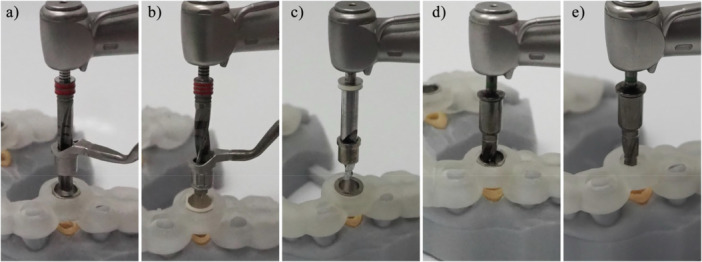
Designs of drills for different Guided Implant Surgery Systems (GISS, C16): (a) Sleeve‐in‐sleeve, non‐interlocking, (b) Sleeve‐in‐sleeve, interlocking, (c) Mounted sleeve‐in‐drill, (d) Integrated sleeve‐in‐drill, (e) Integrated sleeve‐in‐drill, sleeveless surgical guide. Adapted from Sittikornpaiboon et al. (2021).

A CAD/CAM surgical guide which is designed without the use of an additional sleeve in the sleeve tube of the surgical guide. In such designs, the surgical guide directly interfaces with other elements of the surgical kit, such as drilling keys or implant drills, during implant osteotomy and placement (Figure 3e).

*Syn*: Sleeveless surgical guide

**11. Open‐sleeve surgical guide**

A CAD/CAM surgical guide which is designed without the use of an additional sleeve in the sleeve tube of the surgical guide. In such designs, the surgical guide directly interfaces with other elements of the surgical kit, such as drilling keys or implant drills, during implant osteotomy and placement.

**12. Drill handle**

A device that interfaces with the surgical kit components and the surgical guide, allowing the use of drills with varying diameters for implant osteotomy. Drill handle is typically used in a sleeve‐on‐sleeve s‐CAIS systems (Figure 3a,b).

**13. Guided drill**

Implant osteotomy drills for use under s‐CAIS approach, interfacing with other components of the surgical kit and/or surgical guide. Such drills are typically longer and have specific guide elements and markings. Their design can vary significantly depending on the Guided Implant Surgery system they belong to.

**14. Guided Implant Surgical Kit**

A set of drills and components specially designed for conducting the implant osteotomy and implant placement through the s‐CAIS approach. Such kits typically include specially designed instruments, that interface with the components of the surgical guide, such as drills with guide elements and length markings, drill handles and guided implant transfers.

*Syn*: Guided Implant Surgery set, Guided Implant Surgery cassette

**15. Guided Implant Surgery Protocol**

*Abr*: GISP

The surgical protocol prescribed for the use of a Guided Implant Surgical System. The protocol describes the selection, correct sequence and handling of individual components of a Guided Implant Surgery System during the implant surgery.

**16. Guided Implant Surgery System**

*Abr*: GISS

A set of devices and components specially designed for the preparation of the osteotomy and the final placement of the implant with the s‐CAIS approach. Several Guided Implant Surgery Systems are commercially available by different manufacturers, and they do not have interoperability.

**17. Sleeve on sleeve**

*Abr*: SoS

The Guided Implant Surgery System (GISS) which utilizes a handheld drill handle in addition to the surgical guide sleeve for guiding the drills and the implant placement. The drill handle reduces the diameter of the sleeve to the actual drill diameter, while it also controls the vertical stop of the guided drill. Sleeve on sleeve can come two forms: (a) interlocking, where the rotation of the drill handle in the sleeve is not permitted and (b) non‐interlocking, where the drill handle can rotate freely in the sleeve (Figure 3a,b).

*Syn*: Drill‐key system

**18. Mounted sleeve‐on‐drill**

*Abr*: MSoD

The Guided Implant Surgery System (GISS) which utilizes a cylinder attached to each guided drill, matching the internal diameter of surgical guide sleeve. The MSoD GISS utilizes no drill handle and the vertical stop is typically controlled by the surgical guide sleeve (Figure 3c).

**19. Integrated sleeve on drill**

*Abr*: ISoD

The Guided Implant Surgery System (GISS) which utilizes guided drills with an integrated cylindrical “barrel” shape, interfacing with the internal diameter of the surgical guide sleeve. The ISoD GISS utilizes no drill handle and the vertical stop is typically controlled by the surgical guide sleeve (Figure 3d, e).

*Syn*: Keyless systems

**20. Guided implant transfer**

Device attached to a dental implant which connects to a handpiece of a handheld wrench and facilitates the guided placement of the implant through a surgical guide.

*Syn*: Guided implant driver – mount.

**21. Milling**

Subtractive CAM technology of creating 3‐dimensional objects by removing material from standardized blocks by rotating cutting tools.

Adjusted from Glossary of Prosthodontic terms 10th edition (The Glossary of Prosthodontic Terms 2023: Tenth Edition)

**22. Sleeve**

A cylindrical component of the CAD/CAM surgical guide, typically made of metal (e.g., surgical‐grade stainless steel, titanium), PEEK, or zirconia, embedded in or attached to the sleeve tube of the surgical guide. This component interfaces with other elements of the surgical kit, such as drilling keys or implant drills, during implant osteotomy and placement.

**23. Surgical guide resin**

Specialized medical grade plastic material used in CAM manufacturing and 3D printing of dental surgical guides. It must be autoclavable and biocompatible.

**D. dynamic or navigation Computer‐Assisted Implant Surgery**

**1. d‐CAIS System or Navigation System**

A system designed to provide real‐time guidance of surgical instruments used for osteotomies, implant site preparation and implant placement. Optical trackers are rigidly attached to the surgical handpiece and patient, which remain within the line of sight of stereoscopic cameras. The navigation system algorithm triangulates the optical trackers continuously, to determine their precise position and orientation in a common coordinate frame during the surgery. Guidance information is displayed in real‐time on a digital display to assist the osteotomy and implant placement according to the patient‐optimized implant position (see B16). d‐CAIS systems typically include (a) spatial positioning system (see E14), (b) display system, (c) control system (Figure 10).

**2. Fiducial marker ‐ optical**

A device or structure which can be used to relate a spatial point with a point in an imaging technique, such as a CBCT. In CAIS settings it typically refers to an object placed in the field of view of an Optical Tracking System designed to allow rapid, low‐latency detection of 6D position (3D location and 3D orientation).

*Syn*: optical tracker, optically tracked marker, tracking array

**3. Fiducial marker – radiographic**

Radio‐opaque object with a specific shape that is automatically detected by the CAD‐IPS of the d‐CAIS system to perform a radiographic marker‐based patient registration (see D17).

**4. Instrument Registration device**

A device with optical markers or shapes that will be detected by the cameras of the d‐CAIS or r‐CAIS system, serving for the registration of (a) handpiece and (b) drill bits piezoelectric tips or other instruments attached to the contra‐angle handpiece (Figure 5a–e).

*Syn*: Registration plate

Not to be confused with: calibration (see B7)

**Figure 4:**
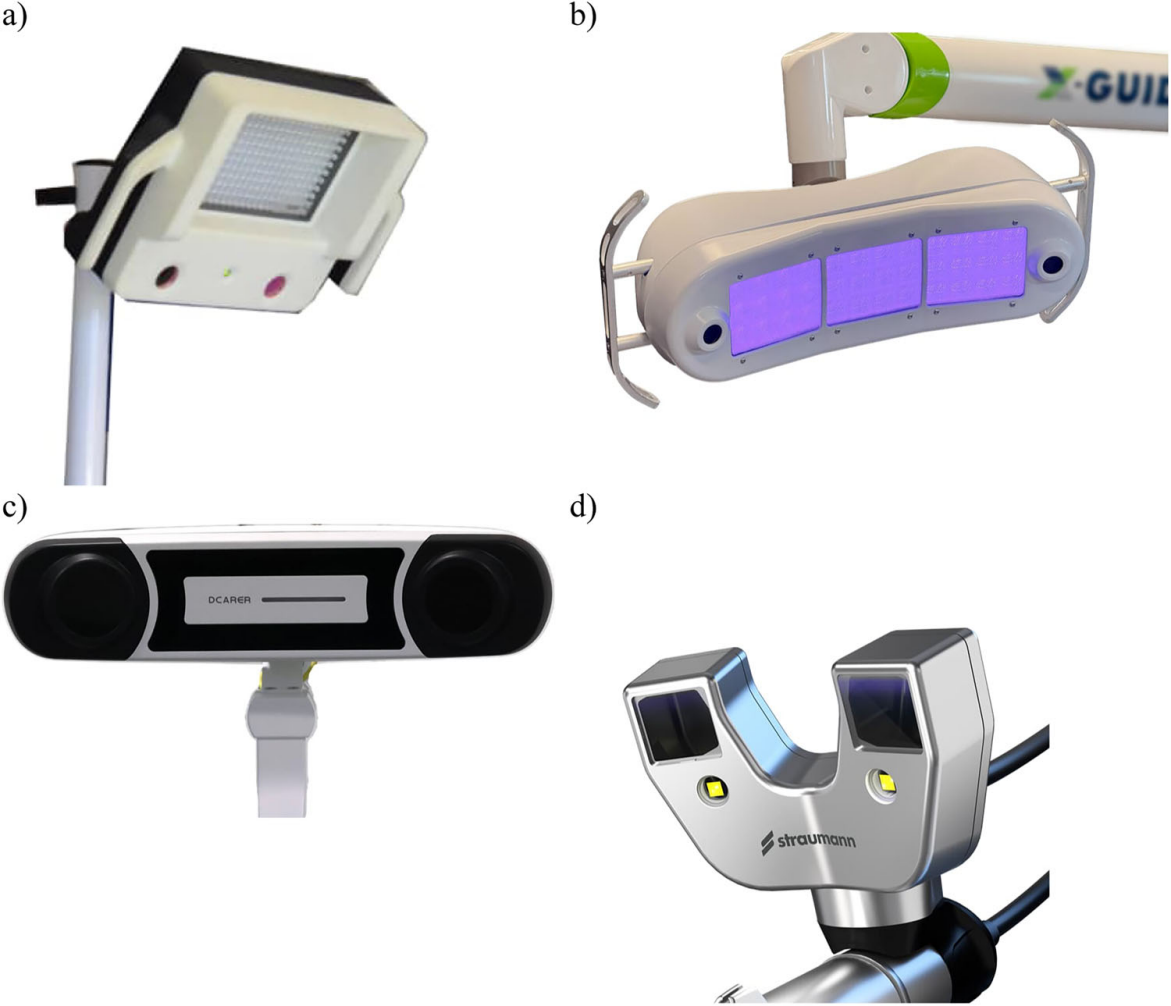
Different commercially available stereoscopic cameras, as part of d‐CAIS systems: (a–c) mounted above or at the side of the surgical field, (d) mounted on the handpiece.

**Figure 5:**
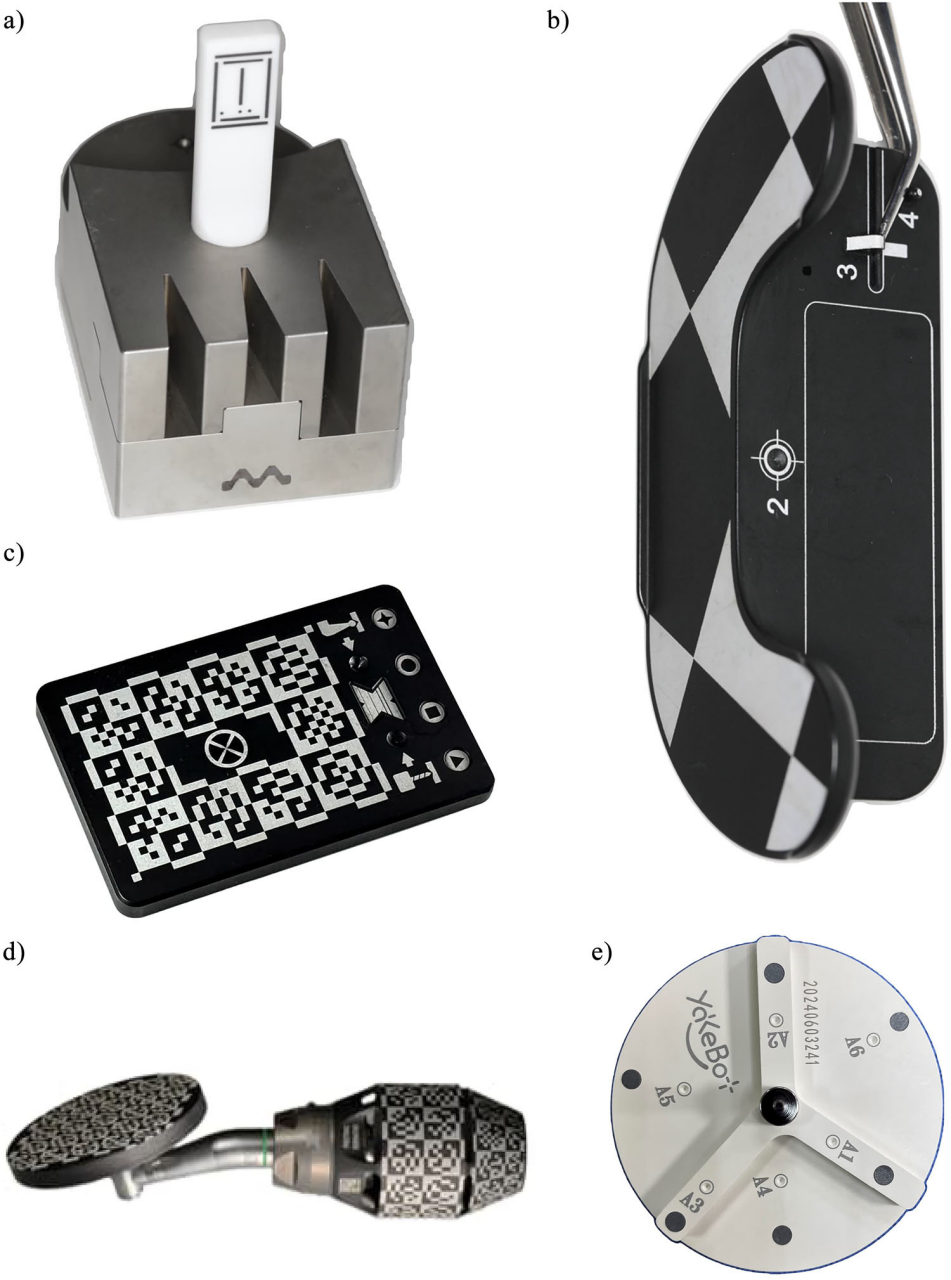
Different commercially available devices to assist instrument registration: (a–c) drill bit registration in a d‐CAIS system, (d) handpiece registration in a d‐CAIS, and (e) in a r‐CAIS system.

**5. Optical Tracking system**

A spatial positioning system (see E14) designed for real‐time spatial tracking of instruments used within a physical space, as well as position and orientation of the patient utilizing optical technology and stereoscopic cameras. The system is typically composed of (a) stereoscopic cameras Figure 4, (b) sets of optical trackers or markers and can be active or passive.

*Syn*: Optical‐, Vision‐based‐Spatial Positioning System.

**6. Active optical tracking system**

Active tracking d‐CAIS systems define the position by detecting the emission of infrared light from devices attached to the surgical instruments and/or the patient.

**7. Passive optical tracking system**

Passive tracking d‐CAIS systems define the position by detecting the reflection of light produced by a light source located next to the camera (infrared or visible), on the markers/trackers attached to the surgical instruments and/or patient.

**8. Optical marker/tracker**

**Figure 6:**
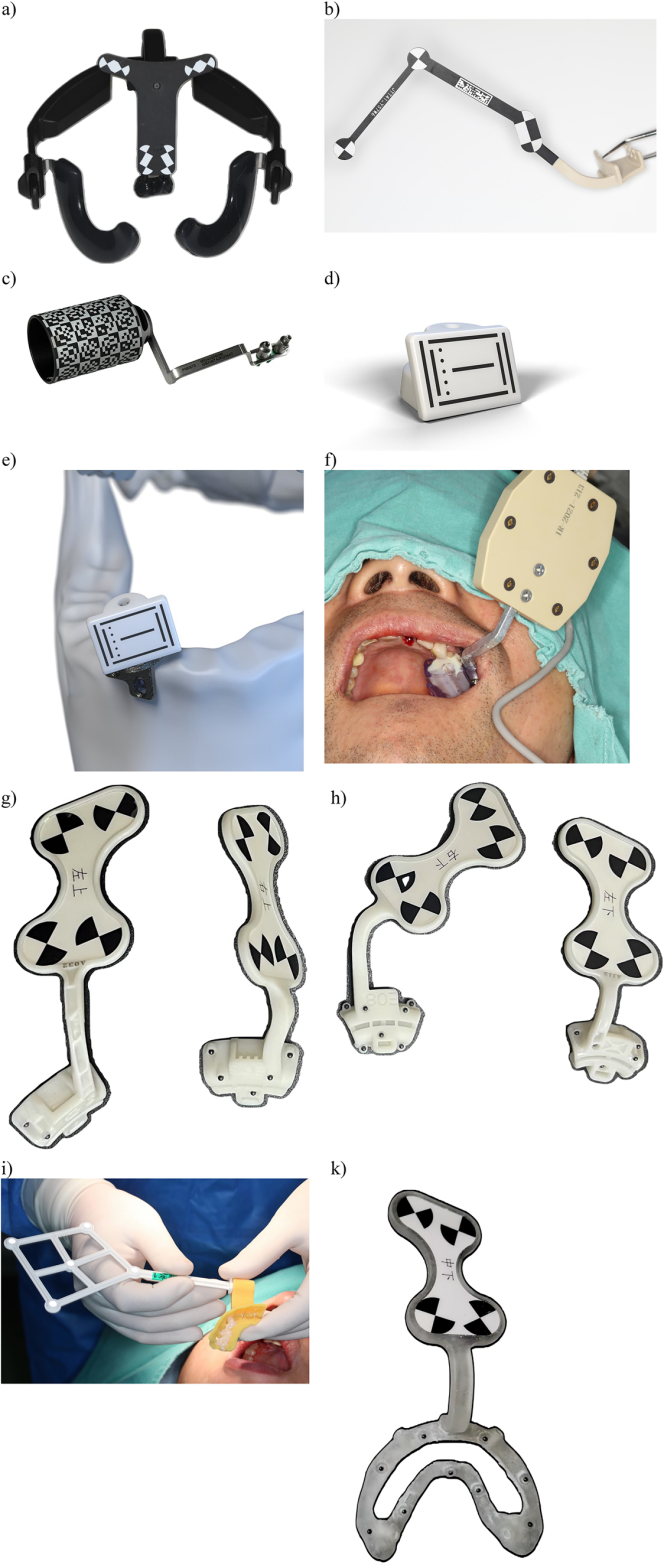
Different commercially available patient optical markers/trackers: (a–e) for passive optical tracking systems, upper and lower jaw, (f) for active optical tracking system, (g–k) for passive optical tracking r‐CAIS system, and (k) bone supported (fixated) tracker for passive optical tracking r‐CAIS system.

**Figure 7:**
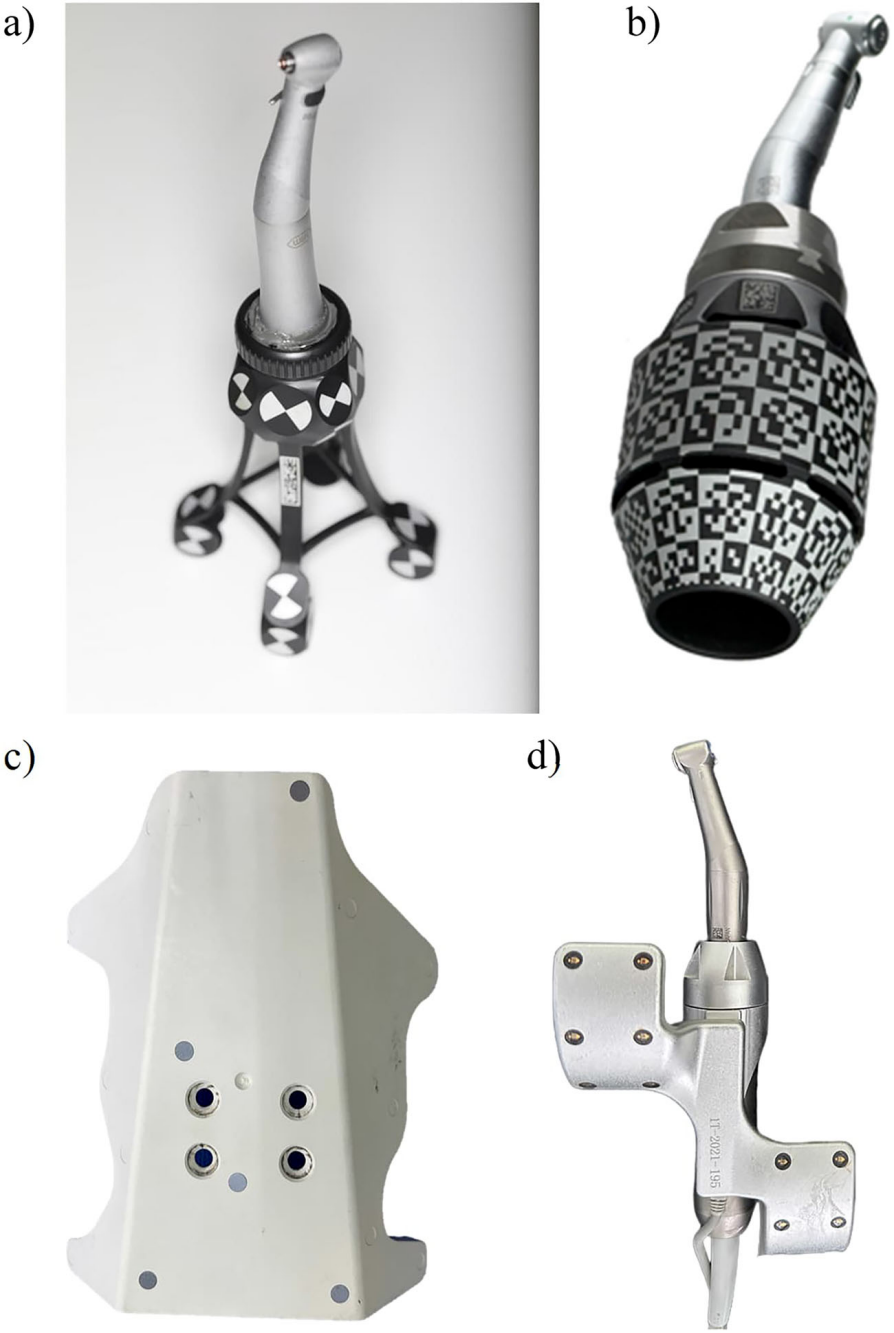
Different commercially available handpiece optical markers/trackers (D9) with different optical fiducial marker (D2) configurations. (a–c) Trackers from passive systems (D7), (d) tracker from active system (D6), (c) tracker for a r‐CAIS system.

A physical device with specific design and shapes which can be detected by the d‐CAIS cameras and serve as reference for the d‐CAIS system. Markers/trackers can be used in either passive or active tracking systems and are typically rigidly attached to the (a) patient (Figure 6a–k) and (b) handpiece (Figure 7a–d).

*Syn*: optical marker, optical tracker, optical tracer, tracking array, optically tracked marker, dynamic reference frame, reference array.

**9. Optical marker/tracker – handpiece**

An optical marker/tracker rigidly attached to the handpiece, allowing the d‐CAIS system to track the position of the contra‐angle handpiece in real time and project it in relation to patient's anatomic structures on a digital display.

**10. Optical marker/tracker – patient**

An optical marker/tracker rigidly attached to the patient's jaw, typically by being mounted on an acrylic stent. The tracker stays in place during the surgery, allowing the d‐CAIS system to track the position of the patient in real time and project the related anatomy on a digital display.

**11. Probe with optical marker/tracker**

**Figure 8:**
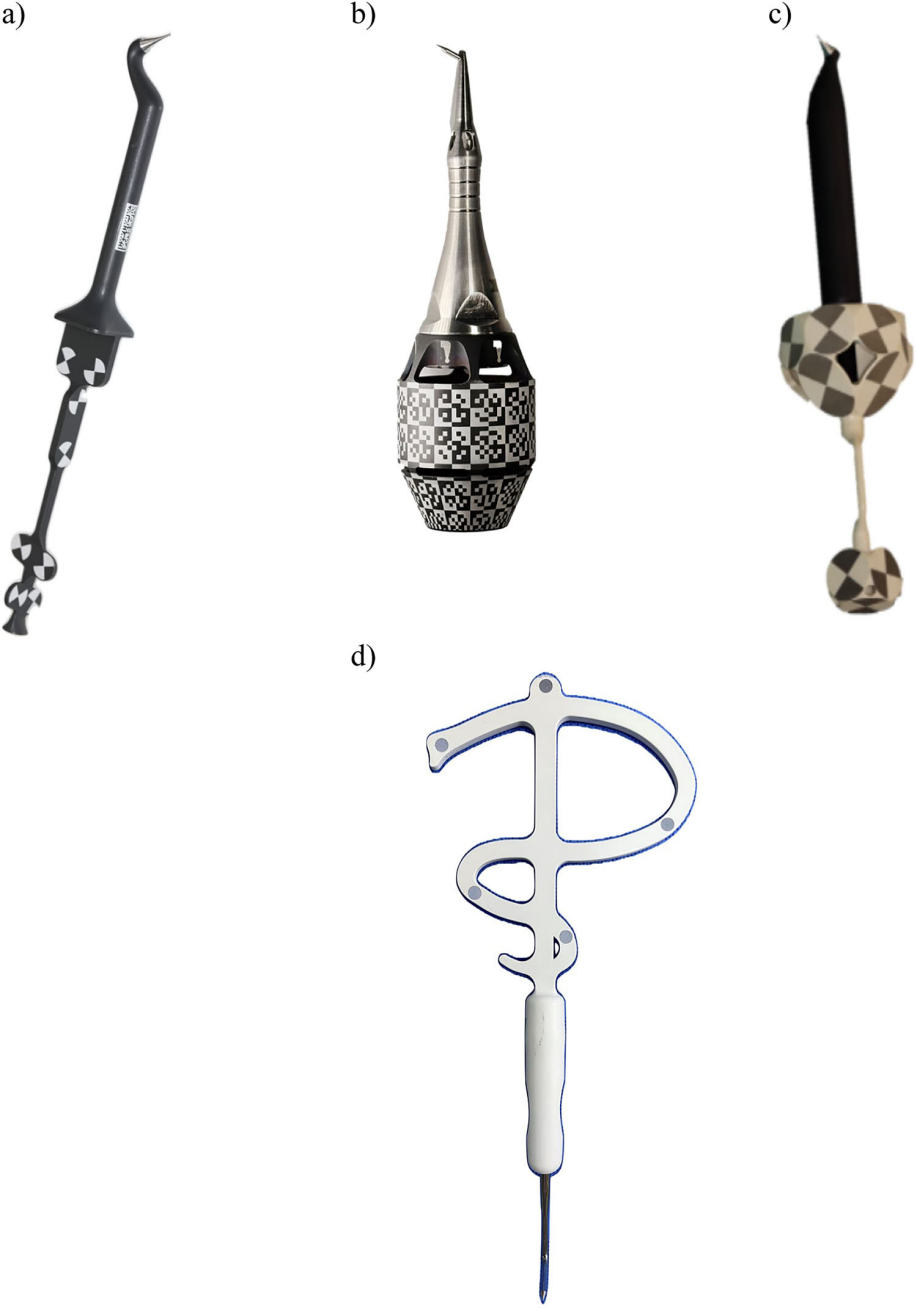
Different commercially available probes with attached optical fiducial markers: (a–c) probes of d‐CAIS systems, (d) probe of r‐CAIS system.

A device with a sharp end like a probe firmly attached to optical tracker or with an embedded optical fiducial marker. It can be used during patient registration in a d‐CAIS or a r‐CAIS system, by matching points in the patient's arch with the corresponding anatomic points in the system (CBCT projected on screen – see D18) (Figure 8a–d).

*Syn*: Optical tracer

**12. Registration**

The process of aligning actual patient's anatomic landmarks or a physical instrument (e.g., drill bit, probe) with their corresponding 3D object in a system of 3‐dimensional coordinates. Registration implies the essential “mapping” of an actual item dimensions and real‐time position within a 3‐dimensional coordinate system, such as the one created and maintained by the spatial positioning system of a navigation device. Registration allows the precise tracking of the instruments during navigation or robotic surgical procedures. Common registration procedures include (a) patient registration, (b) handpiece registration, and (c) instrument registration.

Not to be confused with: calibration (see B7)

**13. 3D data file registration** (see B1).

**14. Handpiece registration**

The process of aligning the surgical handpiece with its corresponding 3D object in the 3‐dimensional coordinates of spatial positioning system, which includes the anatomy of the patient and the digital treatment plan with the virtual implant position.

**15. Instrument registration**

The process of aligning instruments (e.g., drill bits, piezoelectric surgery tips, probes) with their corresponding 3D objects in the 3‐dimensional coordinates of the spatial positioning system, which includes the anatomy of the patient and the digital treatment plan with the virtual implant position.

**16. Patient registration**

The process of aligning the actual patient's anatomy with its corresponding 3D object deriving from the CBCT in the 3‐dimensional coordinates of the spatial positioning system, which includes the treatment plan and the virtual implant position. Patient registration can be made possible by means of different protocols and involve a variety of technologies in different d‐CAIS and r‐CAIS systems.

17. **Radiographic marker‐based patient registration**

The “image to patient” registration protocol, in which the CAD‐IPS of the d‐CAIS system automatically detects a radiographic marker which was attached to the patient's jaw during the CBCT scan.

**18. Pair‐point registration**

The “image to patient” registration protocol in which a set of fiducial points (see D3) or landmarks (typically on the cusps and edges of the remaining teeth) are selected from the CBCT images. These points are then traced on the actual patient's anatomy using a probe with optical tracker (see D11). This approach can be markerless if anatomic landmarks are used as fiducial points, or radiographic marker‐based if, for example, mini‐screws or brand‐specific devices are employed.

**19. Pair‐point and surface registration**

Image to patient registration protocol similar to the pair‐point registration (D18), where after selecting and tracing the fiducial point, a surface surrounding the point is also being traced with the probe to enhance registration accuracy.

**20. Digital marker‐based patient registration**

Image to patient registration protocol, where the optical marker is virtually designed and placed on the jaw during the digital treatment plan in the CAD‐IPS, then physically manufactured through 3D printing without the need of radiographic markers.

**21. Registration Errors**

Errors encountered during the registration process with Optical Tracking Systems (see D5). Three such errors are studied in the literature: (a) Fiducial Localization Error (FLE), (b) Fiducial Registration Error (FRE), and (c) Target Registration Error (TRE) which is the same as FRE but computed on an actual target point different from the fiducials, e.g., a location of interest on the patient. This quality makes the TRE often the most clinically relevant of the three errors.

*Syn*: Tracking Errors

**22. Fiducial Localization Error**

*Abr*: FLE

An error which occurs when the selected marker point in the imaging data is different to its actual location on the patient's anatomy, influenced by either the technical limitations of the imaging system, the design of registration markers or both.

*Syn*: location error

**23. Fiducial Registration Error**

*Abr*: FRE

Fiducial Registration Error (FRE) is defined as to the root mean square value of the distance between corresponding marker points in the virtual coordinate systems and the actual position after registration. It is a critical metric influenced by marker localization accuracy, registration algorithm choice, and image quality.

**24. Target Registration Error**

*Abr*: TRE

Target Registration Error (TRE) represents the discrepancy between a selected point outside the fiducial marker the virtual coordinate system and its actual corresponding position after the registration process. It is influenced by various factors such as the number of landmarks, the position of landmarks, and FLE.

**25. Stereoscopic cameras**

*Abr*: Stereo camera

Camera device with two optical sensors designed to simulate binocular vision capturing two offset images (one for each camera) to create a three‐dimensional image by means of triangulation (Figure 4).

**26. Visible light stereoscopic cameras**

A stereoscopic camera system operating in the visible light spectrum (approximately 380–700 nm) which captures color information along with three‐dimensional spatial data. Their performance may be affected by variations in ambient illumination, shadows, and reflections from surgical instruments.

**27. Infrared stereoscopic cameras**

A stereoscopic camera system operating in the infrared spectrum (typically 850–940 nm) used in d‐CAIS for real‐time tracking.

These systems detect either passive reflective markers or active IR‐emitting markers with high precision, largely unaffected by ambient surgical lighting conditions.

**Figure 9:**
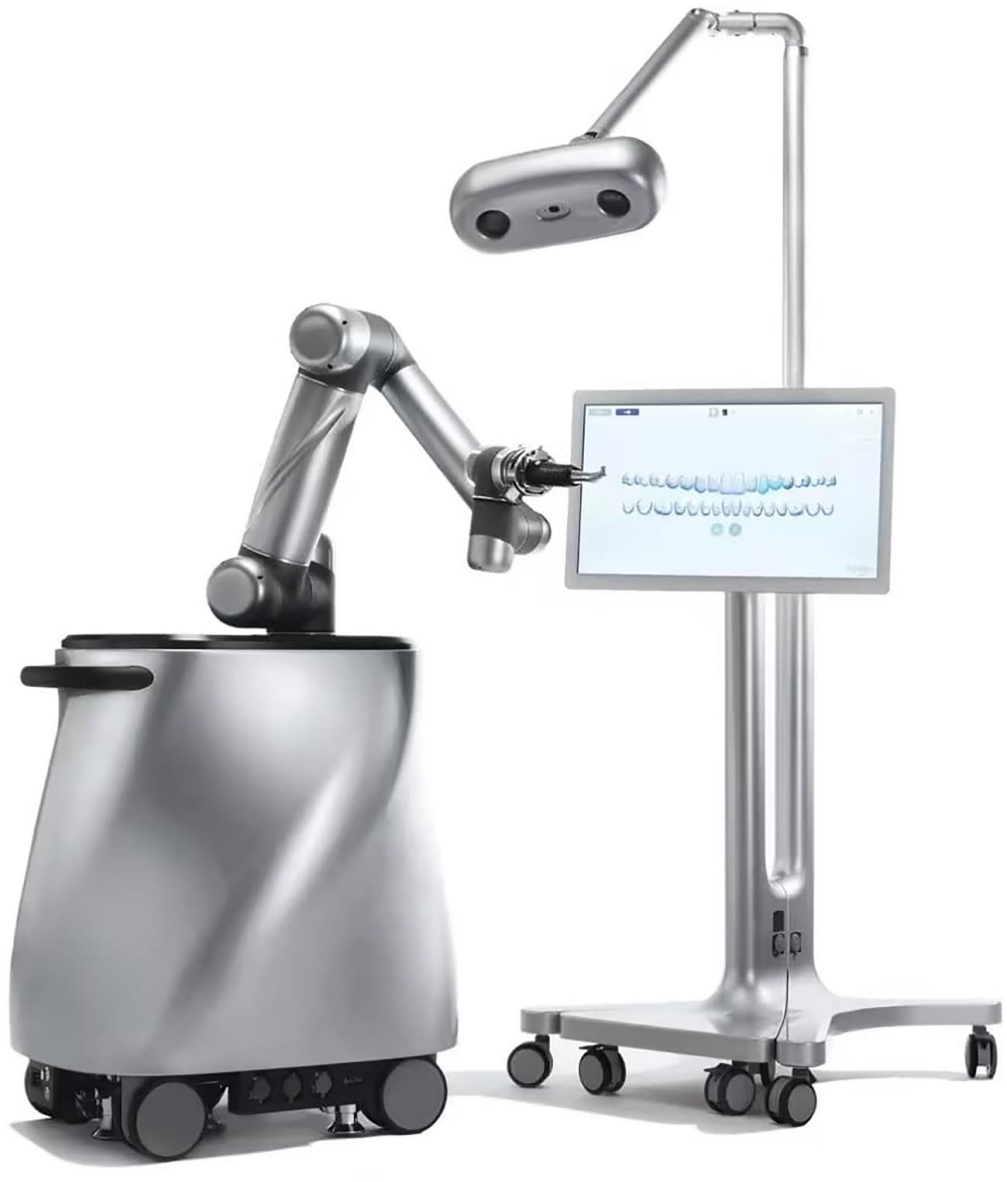
A typical semi‐autonomous CAIS robot. Portable trolley harboring the main processor, with mounted robotic servo and control system, with the mounted robotic arm and actuator (left), optical 3D spatial positioning system, and mounted display (right). (Photo courtesy YakeBot [Beijing] Technology Co. Ltd.).

**E. Robotic Computer‐Assisted Implant Surgery**

**1. Autonomy Level in Robotic Surgery**

Autonomy of a robotic system describes the extent of the ability of the system to operate and perform procedures or tasks independent of human direct control or supervision. Autonomy of medical robots has been classified in six levels: 0 – no autonomy, 1 – robot assistance, 2 – task autonomy, 3 – conditional autonomy, 4 – high autonomy, 5 – full autonomy (Yang et al. 2017). Robots used in r‐CAIS can be categorized into two autonomy levels: collaborative and task‐autonomous.

**2. Collaborative robots** (robot assistance, autonomy Level 1) necessitate human operators to maintain constant control of the system while executing tasks, and may offer some mechanical guidance or support.

**Figure 10:**
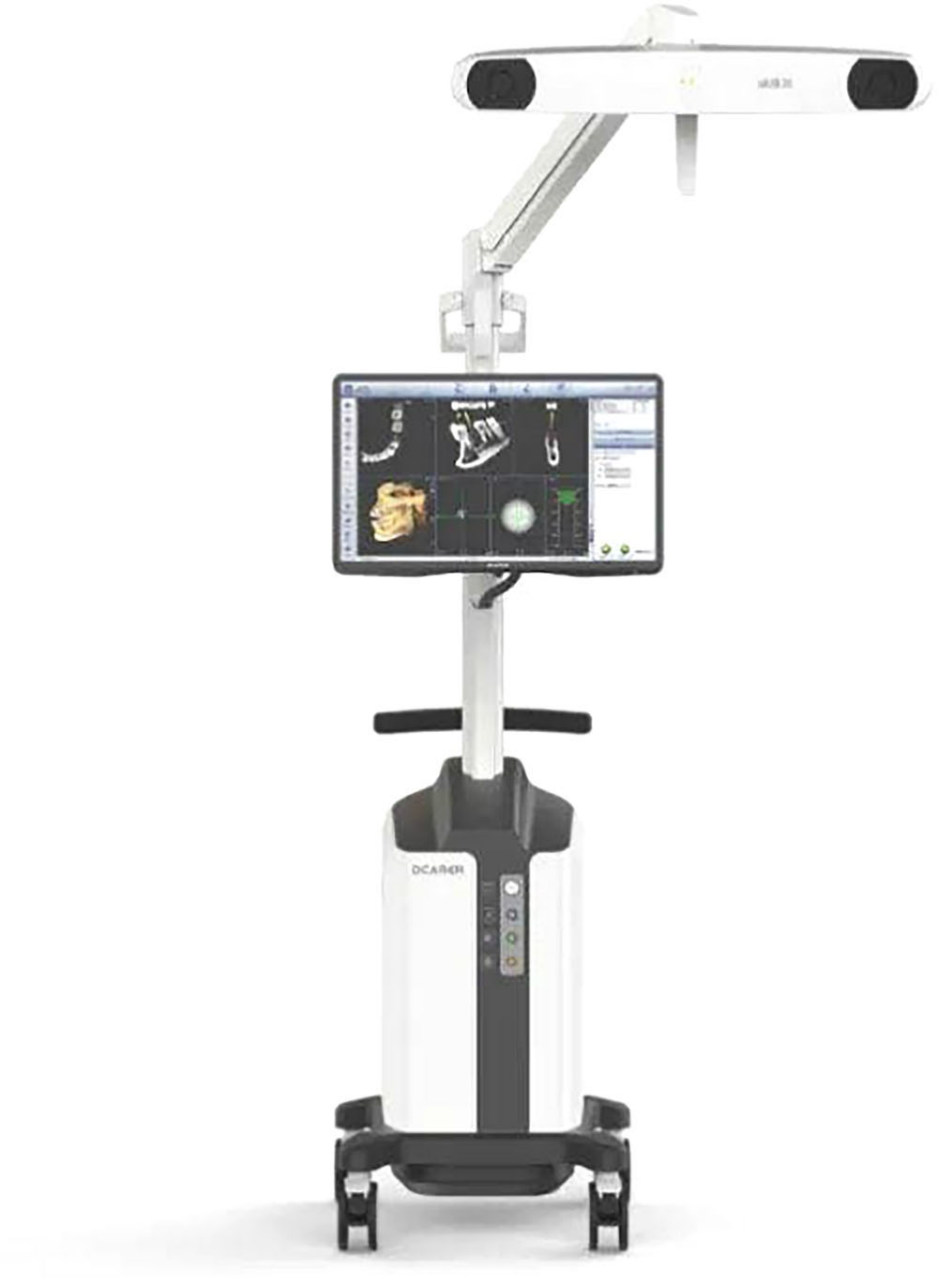
A typical dynamic‐CAIS system, including a portable trolley harboring the main processor, with mounted optical 3D spatial positioning system and display. (Photo courtesy DCarer, Suzhu Digital Health‐care Co. Ltd.).

**3. Task‐autonomous robots** (task autonomy Level 2) only require human operators to exert discrete control over the system, allowing the robot to independently carry out specific, operator‐initiated tasks (Figure 10).

**4. Computer‐Assisted Implant Surgery Robot**

*Abr*: CAIS robot

A specialized medical robot intended to conduct or assist in the execution of tasks during dental implant surgery, such as preparation of the osteotomy and/or placement of the dental implant. CAIS robots typically include (a) spatial positioning system (see E), (b) display system, (c) control system, and (d) actuation system (Figure 9).

*Syn*: r‐CAIS system

**5. Degrees of Freedom**

*Abr*: DOF

Degrees of freedom of a robotic arm refer to the number of axes of movement it has, which also depends on the number of its movable joints. Robotic arms will typically have the ability to perform movements with 1–7 degrees of freedom or on 1–7 different axes in a three‐dimensional space. Typically Increasing the DOF enhances the robot's flexibility and extends its range of motion and the complexity of movements it can perform.

**6. End effector**

Device specifically designed for attachment to the manipulator to enable the robot to perform its task. In the case of CAIS robots, the end effector is commonly a contra‐angle handpiece, mounted with the osteotomy drill bits or implant transfer.

**7. Force Sensor**

A device which can detect the extent and direction of forces applied to the end of the robotic arm (e.g. resistance from socket walls) while performing surgery and conduct automatically essential adjustments to ensure the accuracy and safety of the surgery.

**8. Haptic Feedback**

Technology which enables robots to perceive resistance when applying force to the external environment and adapt to different working scenaria, improving the efficiency and safety of procedures.

**9. Robot**

A programmed actuated mechanism with a degree of autonomy to perform locomotion, manipulation or positioning. Adjusted from (International Organization for Standardization [ISO] 2021).

**10. Medical Robot**

A robot intended to be used as medical electrical equipment or medical electrical system. Based on their function medical robots are categorized (a) surgical robots, (b) rehabilitation robots, (c) diagnostic robots, (d) laboratory analysis automation, and (e) other robots.

Adjusted from ISO 8373:2021 (International Organization for Standardization [ISO] 2021).

**11. Robotic Arm**

The manipulator of the surgical robot, which performs the respective surgical tasks and consists of interconnected links and joints powered by high‐precision motors. These components enable smooth, controlled, and stable movements, allowing the arm to move, reach and interact with tissues during surgery through the end effector (see E6). Adjusted from ISO 8373:2021 (International Organization for Standardization ISO 2021).

*Syn*: Manipulator, actuating – actuation system

**12. Robotic Control System**

Set of hardware and software components implementing logic and power control, and other functions which allow monitoring and controlling of the behavior of a robot and its interaction and communication with other objects and humans in the environment. Adjusted from ISO 8373:2021 (International Organization for Standardization [ISO] 2021).

*Syn*: Robot controller

**13. Robotic Sensors**

Robotic sensors are devices which produce an output signal in response to a physical phenomenon or condition. This includes tactile sensors, visual sensors, force sensors, proximity sensors, ultrasonic sensors, and auditory sensors. According to the different detection object, sensors can be divided into internal and external.

**14. Robotic Servo System**

The system of the surgical robot designed to precisely control motion, ensuring that the mechanical arm and attached instruments move and function accurately and consistently according to the programming or the surgeon's commands. By receiving input signals, the servo system provides high‐precision control over parameters such as position, speed, and acceleration, enabling delicate operations in complex and confined surgical spaces.

**15. Spatial Positioning Systems**

*Abr*: SPS

A system designed for real‐time spatial tracking of instruments attached to the robotic arm, as well as the position and orientation of the patient. SPS can be categorized into (a) mechanical, (b) optical, (c) image‐guided, and (d) electromagnetic positioning systems.

*Syn*: Real‐time spatial positioning system, tracking system

**16. Mechanical spatial positioning systems**

Spatial positioning system which uses physical, mechanical components like linkages, gears and sensors to precisely determine the 3D position and orientation of an object in space. Such systems often rely on precise measurements of joint angles and distances and force feedback to determine and adjust the robotic arm's position.

**17. Optical spatial positioning system**

See Optical Tracking systems (D5)

*Syn*: Vision‐based positioning system, Optical Tracking system

**18. Image‐Guided Spatial Positioning System**

Spatial positioning system which uses internal imaging data from modalities like kilovoltage image, megavoltage image, computed tomography, magnetic resonance image, positron emission tomography, fluoroscopy, or ultrasound to precisely locate an object or tissue within the body, typically without clear line of sight.

Not to be confused with: Optical Spatial Positioning Systems

**19. Electromagnetic spatial positioning systems**

Spatial positioning system which uses magnetic or electromagnetic fields to determine the 3‐dimensional position and orientation of objects. Typically, a sensor is attached to the object, such as a needle or probe, while the object is within the system's measurement range.

## Author Contributions

Conceptualization: Adrià Jorba‐Garcia and Nikos Mattheos. Data curation: Adrià Jorba‐Garcia, Feng Wang, and Nikos Mattheos. Formal analysis: Adrià Jorba‐Garcia and Nikos Mattheos. Investigation: Adrià Jorba‐Garcia, Alessandro Pozzi, Sofya Sadilina, and Feng Wang. Methodology: Bilal Al‐Nawas, Nikos Mattheos, and Atiphan Pimkhaokham. Project administration: Bilal Al‐Nawas and Nikos Mattheos. Resources: Bilal Al‐Nawas and Nikos Mattheos. Supervision: Nikos Mattheos. Validation: Adrià Jorba‐Garcia, Alessandro Pozzi, Sofya Sadilina, Nikos Mattheos, James Chow, and Feng Wang. Visualization: Adrià Jorba‐Garcia, Nikos Mattheos, and Feng Wang. Writing – original draft: Adrià Jorba‐Garcia and Nikos Mattheos. Writing – review & editing: Alessandro Pozzi, Sofya Sadilina, Bilal Al‐Nawas, Zhuofan Chen, James Chow, Romain Doliveux, Yiu Yan Leung, Katsuhiro Maruo, Atiphan Pimkhaokham, Adam Siu, Kay Vietor, Feng Wang, Yiqun Wu, Man Yi, ITI Network in CAIS [For a complete list of the ITI Network in CAIS, see the Acknowledgments section].

## Conflicts of Interest

The authors declared no conflict of interest. Adrià Jorba‐Garcia, Bilal Al‐Nawas, Alessandro Pozzi, Sofya Sadilina, Zhuofan Chen, James Chow, Romain Doliveux, Yiu Yan Leung, Katsuhiro Maruo, Adam Siu, Kay Vietor, Feng Wang, Yiqun Wu, and Man Yi have received travel grants from the International Team for Implantology (ITI) for attendance of the meetings related to the writing of the Glossary.

## Data Availability

The authors have nothing to report.

## References

[cre270148-bib-0001] 2021. “Glossary of Digital Dental Terms, 2nd Edition: American College of Prosthodontists and ACP Education Foundation.” Journal of Prosthodontics 30, no. S3: 172–181. 34878191 10.1111/jopr.13439

[cre270148-bib-0002] 2023. “The Glossary of Prosthodontic Terms 2023: Tenth Edition.” Journal of Prosthetic Dentistry 130, no. 4 Suppl 1: 7–126. 10.1016/j.prosdent.2023.03.002. 37914441

[cre270148-bib-0003] International Standardization Organization . n.d. Accuracy (Trueness and Precision) of Measurement Methods and Results — Part 1: General Principles and Definitions. ISO 5725‐1:2023(en). https://www.iso.org/obp/ui/#iso:std:iso:5725:-1:ed-2:v1:en.

[cre270148-bib-0004] National Institute of Standards and Technology (NIST) . Calibration. Glossary | CSRC. https://csrc.nist.gov/glossary/term/calibration.

[cre270148-bib-0005] US Food and Drug Administration . n.d. Augmented Reality and Virtual Reality in Medical Devices. https://www.fda.gov/medical-devices/digital-health-center-excellence/augmented-reality-and-virtual-reality-medical-devices.

[cre270148-bib-0006] International Organization for Standardization (ISO) . “ISO 8373:2021 – Robotics – Vocabulary [Internet]. Geneva: ISO; 2021. https://www.iso.org/obp/ui/#iso:std:iso:8373:ed-3:v1:en.

[cre270148-bib-0007] Jung, R. E. , D. Schneider , J. Ganeles , et al. 2009. “Computer Technology Applications in Surgical Implant Dentistry: A Systematic Review.” International Journal of Oral & Maxillofacial Implants 24 Suppl: 92–109. 19885437

[cre270148-bib-0008] Mattheos, N. , I. Vergoullis , M. Janda , and A. Miseli . 2021. “The Implant Supracrestal Complex and Its Significance for Long‐Term Successful Clinical Outcomes.” International Journal of Prosthodontics 34: 88–100. 10.11607/ijp.7201. 33570524

[cre270148-bib-0009] Pedrinaci, I. , A. Hamilton , A. Lanis , M. Sanz , and G. O. Gallucci . 2024. “The Bio‐Restorative Concept for Implant‐Supported Restorations.” Journal of Esthetic and Restorative Dentistry 36: 1516–1527. 10.1111/jerd.13306. 39210698

[cre270148-bib-0010] Puisys, A. , M. Janda , V. Auzbikaviciute , G. O. Gallucci , and N. Mattheos . 2023. “Contour Angle and Peri‐Implant Tissue Height: Two Interrelated Features of the Implant Supracrestal Complex.” Clinical and Experimental Dental Research 9: 418–424. 10.1002/cre2.731. 36988518 PMC10280599

[cre270148-bib-0011] Sittikornpaiboon, P. , S. Arunjaroensuk , B. Kaboosaya , K. Subbalekha , N. Mattheos , and A. Pimkhaokham . 2021. “Comparison of the Accuracy of Implant Placement Using Different Drilling Systems for Static Computer‐Assisted Implant Surgery: A Simulation‐Based Experimental Study.” Clinical Implant Dentistry and Related Research 23: 635–643. 10.1111/cid.13032. 34288341

[cre270148-bib-0012] Yang, G.‐Z. , J. Cambias , K. Cleary , et al. 2017. “Medical Robotics‐Regulatory, Ethical, and Legal Considerations for Increasing Levels of Autonomy.” Science Robotics 2, no. 4: eaam8638. 10.1126/scirobotics.aam8638. 33157870

